# The role of prophylactic antibiotics in hepatitis B virus-related acute-on-chronic liver failure patients at risk of bacterial infection: a retrospective study

**DOI:** 10.1186/s40249-021-00830-7

**Published:** 2021-03-31

**Authors:** Xiao-Qin Liu, Xue-Yun Zhang, Yue Ying, Jian-Ming Zheng, Jian Sun, Wen-Hong Zhang, Ji-Ming Zhang, Yu-Xian Huang

**Affiliations:** 1 Department of Infectious Diseases, Huashan Hospital, Fudan University, 12 Middle Urumqi Road, Shanghai, 200040 China; 2 Department of Critical Care Medicine, Huashan Hospital, Fudan University, 12 Middle Urumqi Road, Shanghai, 200040 China

**Keywords:** Acute-on-chronic liver failure, Hepatitis B virus, Infection, Prophylactic antibiotics

## Abstract

**Background:**

Acute-on-chronic liver failure (ACLF) is characterized by an excessive systemic inflammatory response and organ failure and has high mortality. Bacterial infections (BIs) worsen the clinical course of ACLF and carry a poor prognosis in ACLF patients. The efficacy of third-generation cephalosporins has been challenged in recent years. The aim of this study was to characterize the difference between ACLF patients with and without BIs and to provide a reference for medical intervention.

**Methods:**

A total of 140 patients with hepatitis B virus-related ACLF (HBV-ACLF) hospitalized at the Department of Infectious Diseases, Huashan Hospital, Fudan University (Shanghai, China) between May 2013 and January 2020 were enrolled. Mann-Whitney U test was used to compare the baseline characteristics of HBV-ACLF patients with and without BIs. Univariate and multivariate analyses were performed to find predictors of BIs. The characteristics of BIs and the role of prophylactic antibiotics were profiled.

**Results:**

A total of 97 episodes of BIs occurred in patients during the course of HBV-ACLF. Patients with and without BIs differed in clinical characteristics. The incidence of BIs showed a positive correlation with the ACLF grade (*P* = 0.003) and the clinical course (*P* = 0.003). The 90-day transplant-free survival of patients with BIs was lower than those without BIs (*P* < 0.0001). Patients administered prophylactic antibiotics showed a lower incidence of BIs and had a higher transplant-free survival probability than those who did not (*P* = 0.046). No statistical differences in antibiotic efficacy between third-generation and other antibiotics were observed (*P* = 0.108).

**Conclusions:**

BIs affected the clinical course and prognosis of patients with HBV-ACLF. Prophylactic antibiotics were of potential clinical importance in the prevention of BIs and improving the clinical course and prognosis in HBV-ACLF patients. Third-generation cephalosporins were qualified for use in antibiotic prophylaxis.

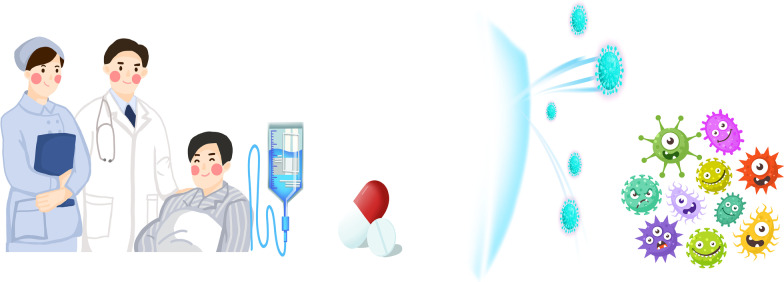

**Supplementary Information:**

The online version contains supplementary material available at 10.1186/s40249-021-00830-7.

## Background

Acute-on-chronic liver failure (ACLF) is a severe clinical syndrome of chronic liver disease and is accompanied by an increased risk for short-term mortality [1, 2]. Chronic hepatitis B infection (CHB) is one of the important etiological factors for HBV-associated acute-on-chronic liver failure (HBV-ACLF) in developing countries. Moreover, HBV-ACLF accounts for 87–91% of overall ACLF cases in China [3, 4]. HBV-ACLF has emerged as a serious healthcare and financial problem in developing countries [5].

Bacterial infections (BIs) are both a precipitating factor and a common complication of ACLF [6, 7]. Previous studies have reported that 45.9–75.5% of ACLF patients who did not acquire BIs at disease onset developed BIs during follow-up due to immunoparesis [6, 8–10]. Furthermore, the occurrence of BIs exacerbated the inflammatory response and aggravated the ischemia–reperfusion injury of organs, such as the liver, kidneys, and lungs, which subsequently resulted in organ failure and an unfavorable prognosis [8, 11]. BIs were an independent predictor of death in patients with ACLF-1 and ACLF-2 [8], and thus, preventing BIs is of great significance for ACLF patients [7, 12]. Prophylaxis for BIs applies to the following situations: (1) after an episode of spontaneous bacterial peritonitis (SBP); (2) patients with variceal bleeding; and (3) patients at high risk of developing bacterial infections [7]. In patients with cirrhosis, prophylactic antibiotic is a vital strategy in preventing not only additional BIs, but also hepatorenal syndrome, recurrent variceal hemorrhage, and even death [7, 12, 13]. However, few studies focused on the role of prophylactic antibiotics in hepatitis B virus-related acute-on-chronic liver failure patients. On the other hand, due to the rise of antibiotic-resistant bacteria, especially multidrug-resistant (MDR) bacteria, the efficacy of third-generation cephalosporins was challenged [7, 13, 14], whereas report about the efficacy of third-generation cephalosporins in HBV-ACLF patients was rare. This study aimed to profile the prevalence of BIs in patients with HBV-ACLF, demonstrate the clinical benefits of prophylactic antibiotics, and provide new medical evidence for specific therapeutic strategies.

## Methods

### Study subjects

In this study, HBV-ACLF patients admitted to the Department of Infectious Diseases, Huashan Hospital, Fudan University (Shanghai, China) from May 2013 to January 2020 were recruited. The inclusion criteria of HBV-ACLF were based on the consensus recommendations of the Asian Pacific association for the study of the liver (APASL) 2019: (1) jaundice (serum bilirubin ≥ 85 mmol/L and coagulopathy [international normalized ratio (INR) ≥ 1.5 or prothrombin activity < 40%]; (2) complicated within four weeks by clinical ascites and/or encephalopathy with previously diagnosed or undiagnosed chronic liver disease/cirrhosis [15]. Chronic hepatitis B was defined by serum hepatitis B surface antigen-positive and/or HBV-DNA-positive for at least 6 months [16].

The exclusion criteria included: (1) patients aged < 18 years; (2) patients with serious underlying diseases of the brain, heart, kidneys, lungs, and other organs; (3) patients with malignant tumors; (4) pregnant women; (5) patients with incomplete clinical data; (6) patients that received a liver transplant; and (7) patients already had definite bacterial infections when diagnosed.

**Fig. 1:**
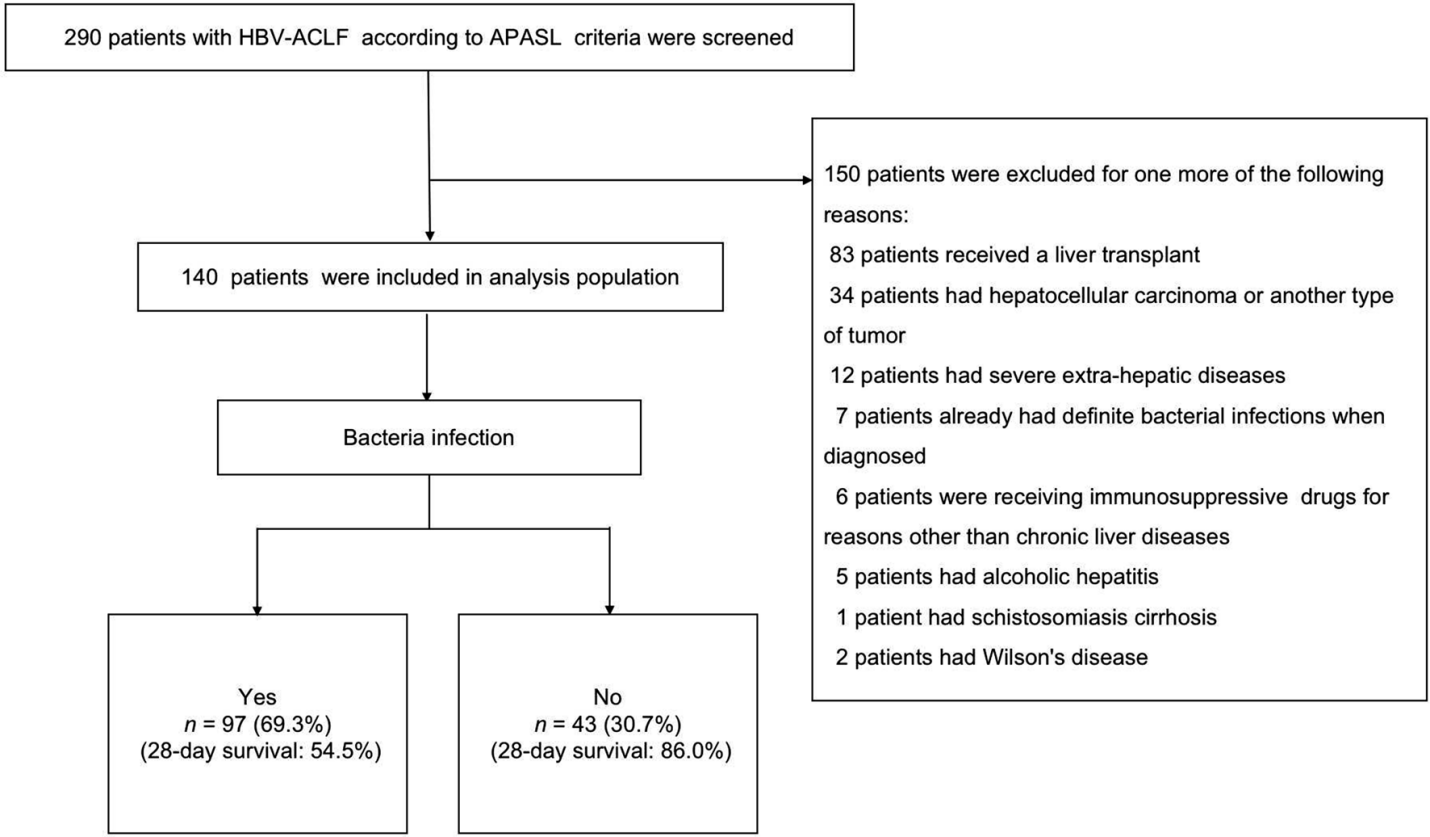
Flow diagram of the patient recruitment process followed in this study. *HBV-ACLF* Hepatitis B virus-related acute-on-chronic liver failure; *APASL* Asian Pacific Association for the Study of the Liver

Finally, a total of 140 patients satisfied the inclusion criteria and 150 patients were excluded (Fig. 1).

### Definitions related to bacterial infection

BI episodes were recorded during the course of HBV-ACLF and diagnosed based on the following criteria. Bacterial peritonitis was diagnosed as a polymorphonuclear cell count greater than 250/mm^3^ in ascites, with or without a positive ascites culture [17]. Pneumonia was diagnosed by the presence of radiological evidence of consolidation plus at least two of the following criteria: fever higher than 38 °C or hypothermia less than 36 °C, cough, pleuritic chest pain, purulent sputum, dyspnea, or signs of consolidation upon medical examination [18]. Bloodstream infection was diagnosed by the growth of a non-common skin contaminant from ≥ 1 blood culture and of a common skin contaminant from ≥ 2 blood cultures drawn at separate sites with signs of infection [19]. Biliary tract infection was diagnosed by a detailed examination, including consultation and physical examination, after which blood tests and diagnostic imaging were performed [20]. Unproven BIs were diagnosed by the presence of fever and leukocytosis, which required antibiotic therapy without any identifiable source [21]. Multi-site infection referred to a situation where two or more infections mentioned above occurred simultaneously. Other infections referred to a perianal abscess case in this study.

### Definitions related to ACLF grade

The final 140 enrolled patients were graded, according to Chinese Group on the Study of Severe Hepatitis B (COSSH)-ACLF criteria, into four groups [22]:

(i) ACLF grade 0. This group includes patients who fulfilled the criteria of APASL-ACLF but did not meet the criteria of organ failures.
(ii) ACLF grade 1. This group includes four subgroups: (1) patients with kidney failure alone; (2) patients with single liver failure with an INR ≥ 1.5 and/or kidney dysfunction and/or hepatic encephalopathy (HE) grade I or II; (3) patients with a single type of organ failure of the coagulation, circulatory or respiratory systems and/or kidney dysfunction and/or HE grade I or II and (4) patients with cerebral failure alone plus kidney dysfunction.
(iii) ACLF grade 2. This group includes patients with failures of two organ systems;
(iv) ACLF grade 3. This group includes patients with failures of three or more organ systems. Organ failures were defined as follows: liver failure: TB ≥ 12 mg/dL; cerebral failure: HE grade III or IV; coagulation failure: INR > 2.5; circulatory failure: using vasoconstrictors; respiratory failure: a ratio of PaO_2_ of arterial oxygen to FiO_2_ of ≤ 200 or a SpO_2_ to FiO_2_ ratio of ≤ 214; kidney failure: serum creatinine ≥ 2 mg/dL or the use of renal replacement therapy [23].

### Management protocol

Prophylaxis antibiotics were recommended for patients with low-protein ascites (< 1.5 g/dL) at high risk of SBP [17, 24]. In this study, the application of prophylactic antibiotics or not was mainly based on physicians’ clinical experiences. Prophylactic antibiotics, including third-generation cephalosporins, piperacillin-tazobactam, cefoperazone and sulbactam, carbapenem, and combination therapy with enzyme inhibitors, were prescribed according to the clinical experience of the physicians. Treatment including absolute bed rest, sodium restriction, diuretics, lactulose, oral antiviral drugs (entecavir or tenofovir), and paracentesis combined with albumin infusion for ascites, and L-Ornithine L-aspartate salt for HE belonged to conventional therapy [25–27].

### Data collection and follow-up

Demographic data, medical history, clinical parameters, including vital signs and radiology, laboratory, microbiology, and treatment data, were collected when the diagnosis of HBV-ACLF was made and patients were free of BIs then. The lymphocyte-to-monocyte ratio (LMR), neutrophil-to-lymphocyte ratio (NLR), disseminated intravascular coagulation (DIC) score, transplant-free survival probability, and cumulative incidence of BIs were then calculated. The patients were followed 90 days after their first diagnosis of HBV-ACLF. A 90-day transplant-free survival was considered the primary endpoint. Information on prognosis was confirmed through medical records and telephone contact.

### Statistical analyses

Two-tailed *P* values were calculated, and the significance level was set at *P* < 0.05. Student’s *t* test was used to assess the average of a normally distributed continuous variable. A Mann‐Whitney U test was used to assess the continuous variables when assumptions of normality were not met. Kaplan–Meier curves were plotted to display the survival of patients in terms of BIs and prophylactic antibiotics. Multivariate logistic regression was fitted with a forward stepwise selection method using clinically and statistically baseline factors that had been screened in univariate analysis to assess the clinical factors associated with the BIs in HBV-ACLF patients.

## Results

### Baseline characteristics of the patients

HBV-ACLF patients with and without BIs showed systemically different clinical characteristics and laboratory results according to the baseline data. Among the 97 HBV-ACLF patients with BIs, the median age was 46 (interquartile range: 38–56) years. Males (*n* = 84, 86.6%) were the predominant population. The frequency of complications, including ascites and gastrointestinal hemorrhage, was substantially different between patients with and without BIs (78.4% vs 48.8%, *P* = 0.001; 10.3% vs 0%, *P* = 0.031, respectively). The applied probability of prophylactic antibiotics was 67.4% in HBV-ACLF patients without BIs as compared with 21.2% of those with BIs—this difference was significant (*P* < 0.001). The laboratory data showed that HBV-ACLF patients with BIs experienced a worse clinical course, with a higher total bilirubin level (*P* = 0.003) and lower levels of sodium (*P* = 0.006) and suffer a more intense inflammatory response, with a higher white blood cell count (*P* = 0.036) and neutrophil count (*P* = 0.008), as well as NLR (*P* = 0.003). Additionally, patients with BIs suffered more serious coagulation defects with a higher INR level (*P* = 0.001) and DIC score (*P* = 0.001). Moreover, HBV-ACLF patients with BIs were more prone to suffer organ failures, especially liver (*P* = 0.011), coagulation (*P* = 0.004), and circulation (*P* = 0.022) (Table 1).

**Table 1:** Comparison of clinical features and laboratory results between HBV-ACLF patients with and without bacterial infections

	Patients without bacterial infection (*n* = 43)	Patients with bacterial infection (*n* = 97)	*P*-value
Clinical data			
Age (years)	49 (37–56)	46 (38–56)	0.271
Male sex, % (*n*)	100 (43)	86.6 (84)	**0.010**
Underlying disease, % (*n*)			0.995
Chronic hepatitis B	46.5 (20)	47.4 (46)	–
Compensated cirrhosis	32.6 (14)	32.0 (31)	–
Deompensated cirrhosis	20.9 (9)	20.6 (20)	–
Complications, % (*n*)			
Ascites	48.8 (21)	78.4 (76)	**0.001**
GI hemorrhage	0 (0)	10.3 (10)	**0.031**
Glucocorticoids, % (*n*)	30.2 (13)	29.9 (29)	0.968
Prophylactic antibiotics, % (*n*)	67.4 (29)	21.2 (22)	**0.000**
Laboratory data			
Alanine aminotransferase (U/L)	737 (251–1629)	529 (180–1238)	**0.178**
Albumin (g/L)	32 (29–36)	32 (29–37)	0.087
Total bilirubin (μmol/L)	190.3 (133.0–254.1)	270.27 (183.0–342.6)	**0.003**
Creatinine (μmol/L)	65 (55–75)	70 (57–91)	0.136
Sodium (mmol/L)	139.0 (134–140)	135 (131–138)	**0.006**
White blood cell count (10^9^/L)	5.4 (4.37–7.92)	7.08 (5.28–10.51)	**0.036**
Neutrophil count (10^9^/L)	3.58 (2.60–5.92)	5.76 (3.70–7.85)	**0.008**
Hemoglobin (g/L)	133 (121–147)	121 (95–136)	**0.001**
Platelet count (10^9^/L)	98 (74–131)	91 (67–123)	0.308
INR	1.85 (1.70–2.26)	2.25 (1.85–2.75)	**0.001**
LMR	1.93 (1.27–2.74)	1.44 (1.01–2.07)	0.056
NLR	3.03 (2.00–5.49)	5.19 (3.43–8.40)	**0.003**
DIC score	4 (3–5)	5 (4–6)	**0.001**
Organ failure, % (*n*)			
Liver	69.8 (30)	87.6 (85)	**0.011**
Coagulation	18.6 (8)	44.3 (43)	**0.004**
Kidney	14.0 (6)	15.5 (15)	0.817
Cerebral	9.3 (4)	22.7 (22)	0.060
Lung	4.7 (2)	9.3 (9)	0.348
Circulation	4.7 (2)	19.6 (19)	**0.022**
Transplant-free survival probability (%)			
28-day	86.0	54.5	**0.000**
90-day	83.1	43.1	**0.000**

Data are expressed as the median (interquartile range) or percent (number). Bold numbers represent significant difference (*P* < 0.05)

*ACLF* acute-on-chronic liver failure, *HBV* Hepatitis B virus, *HBV-ACLF* Hepatitis B virus-related acute-on-chronic liver failure, *GI* gastrointestinal, *INR* international normalized ratio, *LMR* lymphocyte-to-monocyte ratio, *NLR* neutrophil-to-lymphocyte ratio, *DIC* disseminated intravascular coagulation

### The characteristics of BIs in HBV-ACLF patients

**Fig. 2:**
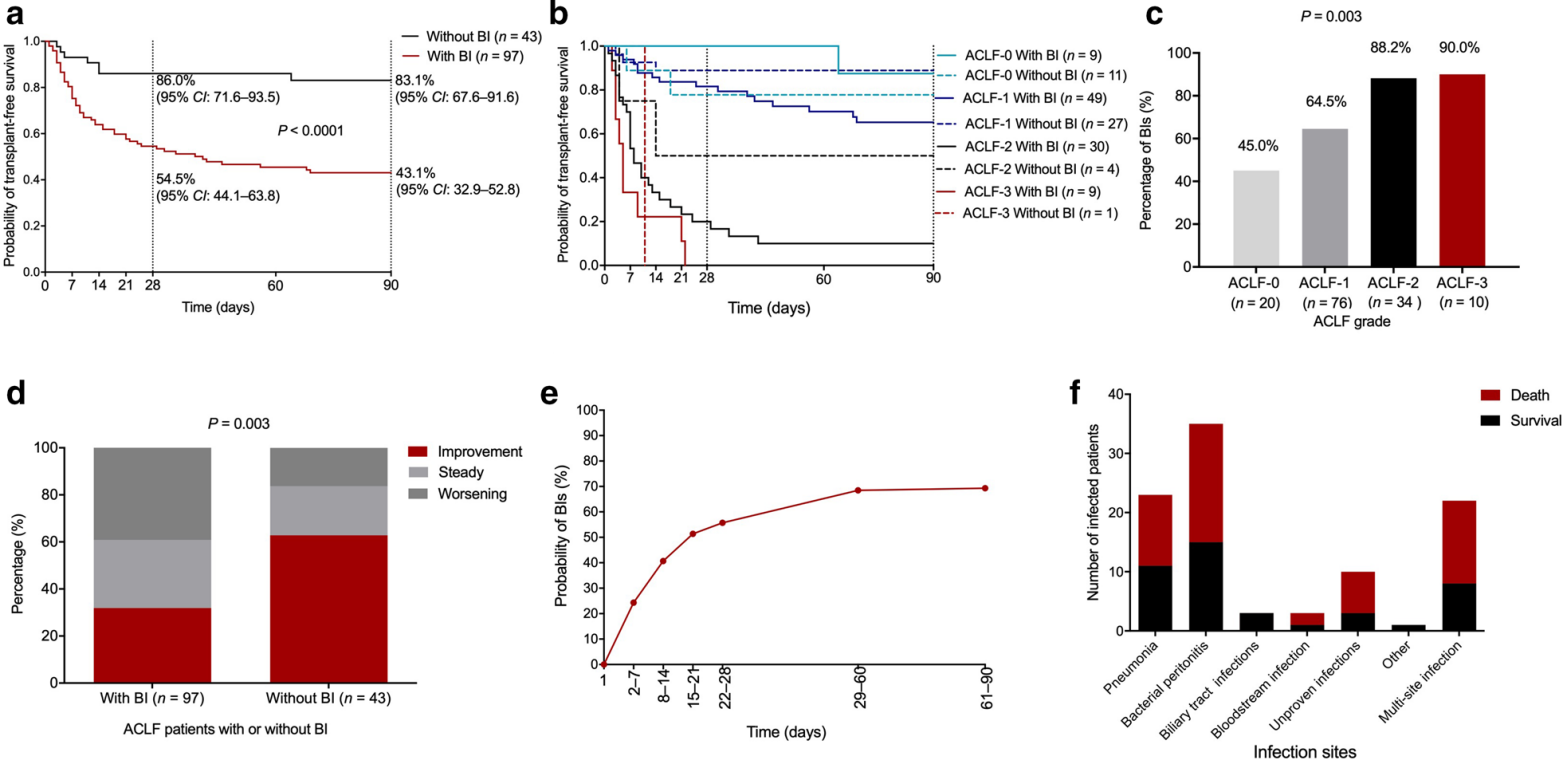
The characteristics of bacterial infections in HBV-ACLF patients. The transplant free survival probability difference between HBV-ACLF patients with and without bacterial infections. **a** In overall study population; **b** in different grades population. **c** The relationship between the incidence of bacterial infections and the severity of HBV-ACLF. **d** The relationship between the occurrence of bacterial infections and the clinical course of HBV-ACLF. **e** The cumulative incidence of bacterial infections among the 140 HBV-ACLF patients within 90-day after their diagnosis of HBV-ACLF. **f** The distribution characters of bacterial infections in HBV-ACLF patients. *HBV-ACLF* Hepatitis B virus-related acute-on-chronic liver failure; *BIs* bacterial infections; *CI* confidence interval

HBV-ACLF patients with BIs had a poorer outcome with a 28-day transplant-free survival of 54.5% and a 90-day transplant-free survival of 43.1% as compared with HBV-ACLF patients without BIs (86.0% and 83.1%, respectively, both *P* < 0.001) (Fig. 2a). The difference was obvious to observe in grade 0, grade 1, and grade 2 HBV-ACLF patients with and without BIs. As for grade 3 HBV-ACLF patients, the transplant-free survival showed no difference (Fig. 2b).

The incidence of BIs increased with the severity of HBV-ACLF (*P* = 0.003). For grades 2 and 3 HBV-ACLF patients, the incidence of BIs increased to 88.2% and 90%, respectively (Fig. 2c). Additionally, HBV-ACLF patients with BIs experienced a worse clinical course than those without BIs and were prone to a poorer hospital outcome (*P* = 0.003) (Fig. 2d). Among all 140 HBV-ACLF patients, the cumulative incidence of infection progressed fastest within the first 28-day course, and the 28-day incidence of BIs was 55.7% in this study (Fig. 2e).

In this study, bacterial peritonitis (36.1%) was the most prevalent infection site, followed by pneumonia (23.7%), multi-site infection (22.7%), unproven infection (10.3%), bloodstream infection (3.1%), biliary tract infection (3.1%), and other infection (1.0%), which referred to one perianal abscess case (Fig. 2f). There were 19 cases of bacteria isolated from 13 patients, including 13 (68.4% of the culture-positive episodes) cases of Gram-negative bacteria and 6 (31.6% of isolates) cases of Gram-positive bacteria. Bacteria were isolated from the ascitic fluid in seven cases, blood in six cases, urine in three cases, and sputum in three cases. Only one bacterium obtained from the urine of a patient without prophylactic antibiotic treatment was confirmed to be MDR *Escherichia coli* (Additional file 1: Table 3).

### Predictors of the incidence of BIs in HBV-ACLF patients

Univariate and multivariate analysis of risk factors for BIs were performed in HBV-ACLF patients. Although univariate analysis showed that sodium [odds ratio (*OR*) = 0.905, 95% confidence interval (*CI*): 0.837–0.979, *P* = 0.013], total bilirubin (*OR * = 1.004, 95% *CI*: 1.001–1.008, *P* = 0.015), hemoglobin (*OR* = 0.974, 95% *CI*: 0.958–0.990, *P* = 0.002), INR (*OR* = 3.484, 95% *CI*: 1.590–7.628, *P* = 0.002), and DIC score (*OR* = 1.486, 95% *CI*: 1.168–1.890, *P* = 0.001) were relevant to the incidence of BIs, these indexes were not predictors in multivariate analysis. Multivariate analysis confirmed that ascites grade (*OR* = 1.802, 95% *CI*: 1.141–2.846, *P* = 0.012), HE grade (*OR* = 2.840, 95% *CI*: 1.015–7.945, *P* = 0.047), and prophylactic antibiotics (*OR* = 0.135, 95% *CI*: 0.056–0.330, *P* = 0.000) were identified as independent predictors of in-hospital BIs in patients with HBV-ACLF. Prophylactic antibiotics was an independent negative prognostic factor in this study (Table 2).

**Table 2:** Predictors of bacterial infection in the univariate and multivariate analyses in patients with HBV-ACLF

Predictor	Univariate analysis	*P*-value	Multivariate analysis	*P*-value
	*OR* (95% *CI*)		*OR* (95% *CI*)	
Age (years)	1.018 (0.989–1.048)	0.233	–	–
Gender	0.000 (0.000–0.000)	0.999	–	–
Underlying liver disease	1.035 (0.402–2.665)	0.932	–	–
Prior decompensation	1.136 (0.456–2.829)	0.784	–	–
Precipitating events	0.935 (0.159–5.495)	0.738	–	–
Total bilirubin (μmol/L)	1.004 (1.001–1.008)	0.015	–	–
Creatinine (μmol/L)	1.005 (0.996–1.013)	0.271	–	–
Sodium (mmol/L)	0.905 (0.837–0.979)	0.013	–	–
White blood cell count (10^9^/L)	1.085 (0.982–1.199)	0.111	–	–
Neutrophil count (10^9^/L)	0.996 (0.999–1.045)	0.870	–	–
Hemoglobin (g/L)	0.974 (0.958–0.990)	0.002	–	–
Platelet count (10^9^/L)	0.995 (0.987–1.002)	0.166	–	–
INR	3.483 (1.590–7.628)	0.002	–	–
**Ascites grade**	**1.951 (1.326–2.871)**	**0.001**	**1.802 (1.141–2.846)**	**0.012**
**Hepatic encephalopathy grade**	**2.528 (1.003–6.371)**	**0.049**	**2.840 (1.015–7.945)**	**0.047**
LMR	0.732 (0.515–1.041)	0.083	–	–
NLR	0.988 (0.959–1.017)	0.402	–	–
DIC score	1.486 (1.168–1.890)	0.001	–	–
Glucocorticoids	0.984 (0.450–2.153)	0.968	–	–
**Prophylactic antibiotics**	**0.142 (0.064–0.314)**	**0.000**	**0.135 (0.056–0.330)**	**0.000**

Bold-face font represents factors that are significant predictors of infection in multivariate analyses

*HBV-ACLF* Hepatitis B virus-related acute-on-chronic liver failure, *INR* international normalized ratio, *LMR* lymphocyte-to-monocyte ratio, *NLR* neutrophil-to-lymphocyte ratio, *DIC* disseminated intravascular coagulation, *OR* odds ratio, *CI* confidence interval

“–” represents factors that are not ultimately included in the multivariate analysis

### The role of prophylactic antibiotics in HBV-ACLF patients

**Fig. 3:**
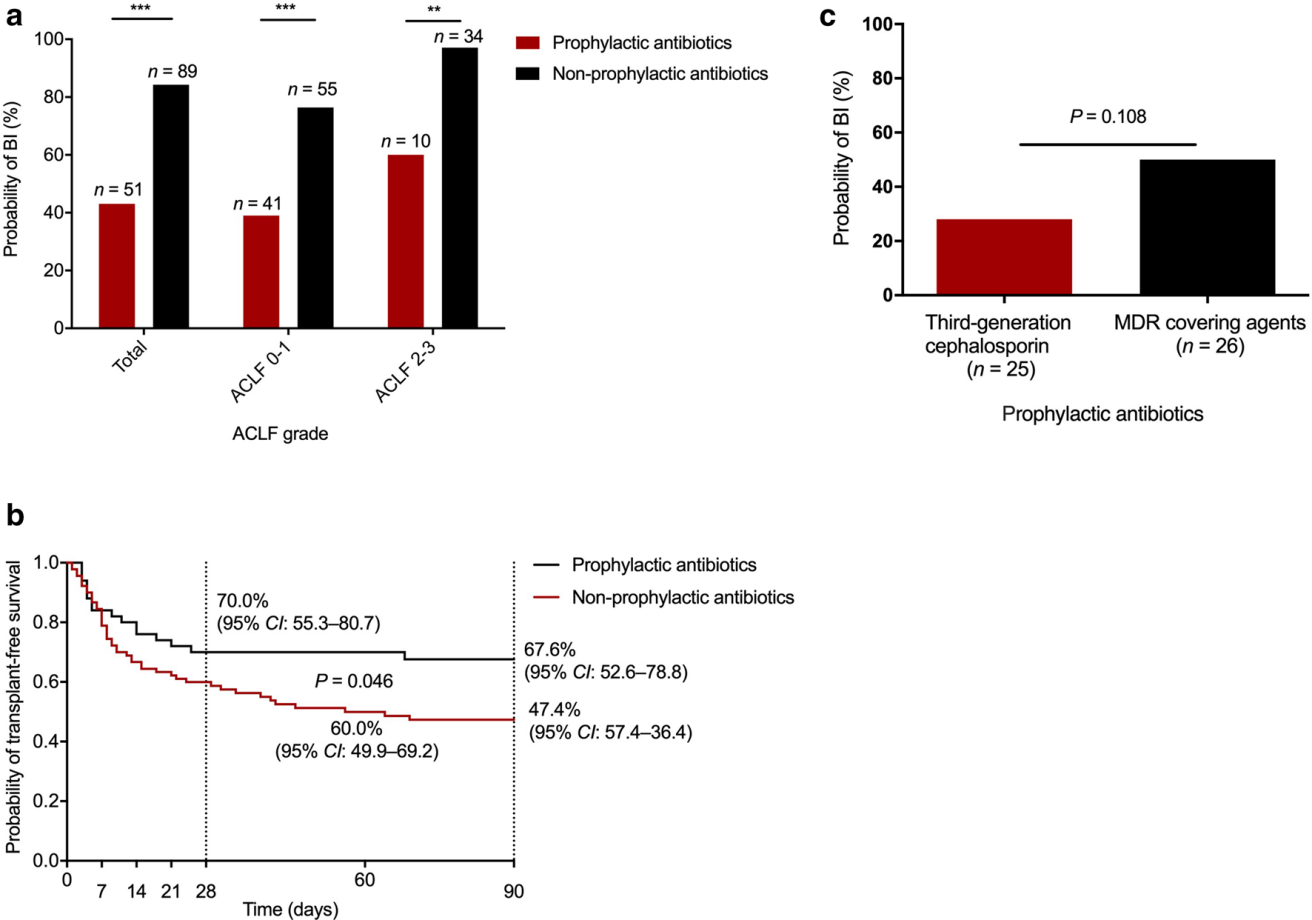
The significance of prophylactic antibiotics on bacterial infections (BIs) and the probability of transplant-free survival. **a** Comparison of the probability of BIs between HBV-ACLF patients with and without prophylactic antibiotics. **b** Comparison of the transplant-free survival probability between HBV-ACLF patients with and without prophylactic antibiotics. **c** Comparison of the probability of BIs between antibiotic regimen with the third-generation cephalosporin and with MDR covering-agents in HBV-ACLF patients. *HBV-ACLF* Hepatitis B virus-related acute-on-chronic liver failure; *MDR* multiple drug resistance; *OR* odds ratio; *CI* confidence interval

Eighty-nine patients received conventional therapy and 51 patients received prophylactic antibiotics in addition to conventional therapy. The median duration of prophylactic antibiotics was 10 (interquartile range: 7–14) days. Patients who did not develop BIs was 7 (interquartile range: 12–15) days and patients who did develop BIs was 5 (interquartile range: 7.5–11.75) days. Third-generation cephalosporins, as a first-line treatment, were administered to 25 patients. MDR-covering agents were also administered to patients, among them six patients received piperacillin-tazobactam, six patients received cefoperazone and sulbactam, eleven patients received carbapenem, and three patients received combination therapy, including enzyme inhibitors and carbapenem. Among the 140 HBV-ACLF patients, those that received prophylactic antibiotics exhibited a lower probability of infection than those without prophylactic antibiotic treatment irrespective of the HBV-ACLF grade level (Fig. 3a). Moreover, patients who received prophylactic antibiotics showed a higher 90-day transplant-free survival than those who did not (67.6% vs 47.4%, *P* = 0.046) (Fig. 3b). As for the efficacy of the antibiotic regimen, there was no statistical difference between third-generation cephalosporins and MDR-covering agents (50% vs 28%, *P* = 0.108) (Fig. 3c). No patients showed side effects of antibiotic treatment in this study.

## Discussion

ACLF is an acute deterioration of chronic liver disease characterized by multiple organ failure and high short-term mortality, with 3-month and 1-year mortality rates being 53.7% and 67.4%, respectively [28]. ACLF patients are highly susceptible to BIs because these patients display sepsis-like immune paralysis [29–31].

BIs play a critical role in the development and progression of ACLF because they exacerbate the inflammatory reaction via pathogen-associated molecular patterns in the body. BIs also trigger the occurrence of ACLF in patients with cirrhosis [1, 8]. The systemic inflammatory response induced by BIs leads to multiple extra-hepatic organ failures and further increases mortality in patients with ACLF [32]. A worse clinical course and a lower 90-day probability of survival (49%) or a higher short-term mortality by two to fourfold was reported in ACLF patients with concurrent BIs (either at diagnosis or during follow-up) than ACLF patients without BIs [8, 33]. Our results also showed that irrespective of the grade level, once BIs occurred, ACLF patients were prone to a lower transplant-free survival than those without BIs. At the same time, our study confirmed that the frequency of BIs increased with higher ACLF grades, varying from 45.0% to 90% (*P* = 0.003). The clinical course was quite different between HBV-ACLF patients with and without BIs. Patients without BIs showed a twofold higher improvement proportion relative to patients with BIs. On the other hand, patients with BIs showed a twofold higher worsening proportion relative to patients without BIs (*P* = 0.003). Thus, in this study, we demonstrated that the occurrence of BIs exerted a negative impact on the severity level, clinical course, and mortality in HBV-ACLF patients, which was consistent with previous studies [6, 8].

As ACLF developed or progressed, the incidence of BIs increased. In this study, 55.7% of patients developed BIs within 28 days after their first diagnosis of HBV-ACLF. Bacterial peritonitis (36.1%) was the most prevalent infection site, followed by pneumonia (23.7%) in this study. Bacterial peritonitis is one of the most common infections in ACLF patients varying from 21.1 to 34.5% in previous studies and is associated with a high risk of developing irreversible renal failure and hepatorenal syndrome [6, 8, 18]. Pneumonia (23.7%) was the second most common infection site in this study, and the frequency of pneumonia varied from 7.7 to 45.0% in previous studies [1, 6, 8]. Bloodstream infection (3.1%) was less common in this study relative to other studies [9, 18]. In our study, the mortality according to different BI sites was 20.6% in bacterial peritonitis, 14.4% in multi-site infection, 12.4% in pneumonia, 7.2% in unproven infection, 2.1% in bloodstream infection, 0% in biliary tract infection, and 0% in other infection.

The increased intestinal permeability was the main cause of bacterial translocation in patients with cirrhosis, And Gram-negative bacteria was the major translocating bacteria [34]. In this study, Gram-negative bacteria remained to be the most frequently isolated bacteria. Only one bacterium (5.3%) obtained from the urine of a patient without prophylactic antibiotic treatment was confirmed to be MDR *Escherichia coli*. Fungi were isolated from three BIs cases, but fungal infections were not evaluated in this study.

In this study, the univariate and multivariate analyses confirmed that ascites grade, HE grade, and prophylactic antibiotics were both independent prognostic factors of BIs. Both ascites grade and HE grade indicated the correlation between the severity level of HBV-ACLF and the prevalence of BIs. Notably, prophylactic antibiotics were an independent negative prognostic factor in this study, which suggested that prophylactic antibiotics protected HBV-ACLF patients from BIs. The positive influence of prophylactic antibiotics was also demonstrated by the improvement in the transplant-free survival probability in this study. Although the severity level of ACLF was closely related to the incidence of BIs, our study further analyzed and found that prophylactic antibiotics played an apparent protective role against BIs, irrespective of the severity of HBV-ACLF as compared with patients without prophylactic antibiotics. This makes sense because the application of prophylactic antibiotics was an important method to limit potential damage and prevent recurrence of infection [11]. However, delayed initiation of antibiotics can lead to the loss of crucial time and worse outcomes in HBV-ACLF patients. Additionally, cirrhosis patients were at high risk of nosocomial infections. Thus, the early administration of prophylactic antibiotics could improve the patient’s prognosis [36].

Several factors, including epidemiological, local microbiological, and risk factors for MDR, need to be considered when it comes to initiating antibiotics [37]. MDR bacterial infections were the main concern for the use of prophylactic antibiotics or inappropriate antibiotics and were usually isolated in the intensive care unit and nosocomial episodes. MDR bacterial infections were serious events related to lower resolution rates and higher short-term mortality [8, 38]. It has been reported that the prevalence of MDR bacterial infections in patients with decompensated cirrhosis and with ACLF increased from 29% to 38% in culture-positive infections from 2011 to 2017–2018 in Europe [39]. However, the MDR rate in our study was lower than other studies [1, 6, 8, 35]. Recent studies have also reported increasing prevalence of Gram-positive bacteria [39–41], while the most frequently isolated bacteria in this study were Gram-negative bacteria, which was consistent with other studies [1, 12].

Previous studies have demonstrated MDR-covering strategies to be effective, as they yield a higher infection resolution rate or a higher chance of microbiological susceptibility as compared to classical antibiotics strategies, especially in nosocomial infections and severe infections [12, 39]. However, in a randomized clinical trial when the infection was not complicated by sepsis, the benefit derived from the use of broad-spectrum antibiotics was blunted [12]. We also need to recognize the toxicity and risk of secondary infection due to the application of broad-spectrum antibiotics. Third-generation cephalosporins have been recommended as an appropriate first-line prophylaxis in advanced cirrhosis patients because of antibiotic effectiveness and low hepatic and renal toxicity [12, 42]. Emerging studies have revealed that the efficacy of third-generation cephalosporins has been reduced due to the spread of MDR bacterial infections [13, 14, 36]. However, evidence has also shown that third-generation cephalosporins, as first-line antibiotics, achieved a recovery rate of approximately 75% in SBP patients and cefotaxime was the first choice in community-acquired infections [6, 43]. As for antibiotic prophylaxis in patients with HBV-ACLF, there was uncertainty about which is most beneficial. Our results also suggested that the antibiotic efficacy of third-generation cephalosporins was higher than that of MDR antibiotics, indicating that third-generation cephalosporins are more appropriate prophylactic antibiotics for HBV-ACLF patients. Although there was no significant difference in the efficacy between third-generation cephalosporins and MDR-covering agents—most likely due to the limited number of patients in this study—further studies are needed to confirm this finding. However, the current need for standardized prophylactic antibiotic therapy for patients with HBV-ACLF was urgent. We need more future randomized clinical trials that were adequately powered, employ blinding to further demonstrated that whether antibiotic prophylaxis is beneficial, and if beneficial, which antibiotic prophylaxis is most beneficial in people with HBV-ACLF.

The major limitation of this study was that in this retrospective study, the application of prophylactic antibiotics or not was mainly based on physicians’ clinical experiences. We recognized that the limitation of retrospective study brought difficulty to popularize the prophylaxis antibiotics in patients, we also looked forward and would be willing to design adequately powered randomized controlled trials to clarify whether the antibiotic prophylaxis has an intervention effect on BIs in HBV-ACLF patients. Mortality and adverse events and other clinically important outcomes should also be evaluated.

## Conclusions

BI is a major risk factor for survival in patients with HBV-ACLF. It is imperative to minimize and prevent the risk of BI. Once ACLF is diagnosed, prophylactic antibiotics should be initiated early to minimize the damage in HBV-ACLF patients. Aside from MDR-covering agents, third-generation cephalosporins are suitable candidates for use in prophylactic strategies.

## Supplementary Information

**Additional file 1: Table 1.** Baseline characteristics of HBV-ACLF patients with and without prophylactic antibiotics. **Table 2.** Baseline characteristics of patients with prophylactic antibiotics. **Table 3.** Characteristics of isolated bacteria in HBV-ACLF patients with bacterial infection

## Data Availability

All data generated or analysed during this study are included in this published article and its supplementary information files (Additional file 1).

## Abbreviations

ACLF: Acute-on-chronic liver failure
APASL: Asian Pacific Association for the Study of the Liver
BIs: Bacterial infections
CHB: Chronic HBV infection
*CI*: Confidence interval
COSSH: Chinese Group on the Study of Severe Hepatitis B
DIC: Disseminated intravascular coagulation
HBV: Hepatitis B virus
HBV-ACLF: Hepatitis B virus-related ACLF
HE: Hepatic encephalopathy
INR: International normalized ratio
LMR: Lymphocyte-to-monocyte ratio
MDR: Multidrug-resistant
NLR: Neutrophil-to-lymphocyte ratio
*OR*: Odds ratio
PTA: Prothrombin activity
SBP: Spontaneous bacterial peritonitis
SOFA: Sequential Organ Failure Assessment
