# Supplementary material for: The role of prophylactic antibiotics in hepatitis B virus-related acute-on-chronic liver failure patients at risk of bacterial infection: a retrospective study
Source: Infect Dis Poverty. 2021 Mar 31;10:44. doi: 10.1186/s40249-021-00830-7 (PMC8011196; doi:10.1186/s40249-021-00830-7)
Supplement: Supplementary file 1 — Additional file 1: Table 1. Baseline characteristics of HBV-ACLF patients with and without prophylactic antibiotics. Table 2. Baseline characteristics of patients with prophylactic antibiotics. Table 3. Characteristics of isolated bacteria in HBV-ACLF patients with bacterial infection [file 40249_2021_830_MOESM1_ESM.docx]

Additional

Table 1. Baseline characteristics of HBV-ACLF patients with and without prophylactic antibiotics

|  | **Patients with prophylactic antibiotics group (*n* = 51)** | **Patients without prophylactic antibiotics group (*n* = 89)** | *P*-value |
| --- | --- | --- | --- |
| **Clinical data** |  |  |  |
| Age (years) | 45 (37-57) | 48 (35-56) | 0.790 |
| Male sex, % (*n*) | 98.0 (50) | 86.5 (77) | 0.024 |
| Underlying disease, % (*n*) |  |  | 0.436 |
| Chronic hepatitis B | 51.0 (26) | 44.9 (40) | - |
| Compensated cirrhosis | 25.5 (13) | 36.0 (32) | - |
| Decompensated cirrhosis | 23.5 (12) | 19.1 (17) | - |
| Complications, % (*n*) |  |  |  |
| Ascites | 68.6 (35) | 74.2 (66) | 0.482 |
| Bacterial infection | 43.1 (22) | 84.3 (75) | 0.000 |
| Hepatic encephalopathy | 17.6 (9) | 15.7 (14) | 0.768 |
| Glucocorticoid, % (*n*) | 37.3 (19) | 25.8 (23) | 0.156 |
| **Laboratory data** |  |  |  |
| Albumin (g/L) | 33 (30-35) | 32 (29-36) | 0.750 |
| Total bilirubin (μmol/L) | 214 (159.2-308.1) | 240.7 (154-343.3) | 0.466 |
| Creatinine (μmol/L) | 66 (55-80) | 70 (57-88) | 0.252 |
| Sodium (mmol/L) | 137 (133-140) | 135 (131-139) | 0.175 |
| White blood cell count (10^9^/L) | 6.14 (4.62-10.51) | 6.63 (4.51-10.14) | 0.993 |
| Neutrophil count (10^9^/L) | 4.4 (2.81-8.54) | 5.03 (3.44-7.36) | 0.714 |
| Hemoglobin (g/L) | 131 (121-146) | 120 (93.5-135.5) | 0.001 |
| Platelet count (10^9^/L) | 100 (84-127) | 89 (60-124) | 0.140 |
| INR | 2.12 (1.76-2.35) | 2.11 (1.84-2.72) | 0.100 |
| LMR | 1.40 (1.05-2.19) | 1.59 (1.05-2.38) | 0.727 |
| NLR | 4.62 (2.29-7.54) | 4.69 (3.00-8.39) | 0.557 |
| MELD | 25 (23-28) | 27 (23-31) | 0.069 |

Abbreviations: HBV-ACLF, hepatitis B virus-related acute-on-chronic liver failure; INR, international normalized ratio; LMR, lymphocyte to monocyte ratio; NLR, neutrophil to lymphocyte ratio; MELD, Model for End Stage Liver Disease; Data are expressed as the median (interquartile range) or percent (number).

Table 2. Baseline characteristics of patients with prophylactic antibiotics

|  | **Third-generation cephalosporins group (*n* = 25)** | **MDR-covering agents**  **group (*n* = 26)** | *P*-value |
| --- | --- | --- | --- |
| **Clinical data** |  |  |  |
| Age (years) | 43 (33-54) | 49 (39-56) | 0.491 |
| Male sex, % (*n*) | 100 (25) | 96.2 (25) | 0.322 |
| Underlying disease, % (*n*) |  |  | 0.972 |
| Chronic hepatitis B | 52.0 (13) | 50.0 (13) | - |
| Compensated cirrhosis | 24.0 (6) | 26.7 (7) | - |
| Decompensated cirrhosis | 24.0 (6) | 23.1 (6) | - |
| Complications, % (*n*) |  |  |  |
| Ascites | 56.0 (14) | 65.4 (17) | 0.493 |
| Bacterial infection | 36.0 (9) | 50.0 (13) | 0.313 |
| Hepatic encephalopathy | 8.0 (2) | 26.9 (7) | 0.140 |
| **Laboratory data** |  |  |  |
| Albumin (g/L) | 33 (30-35) | 33 (30-34) | 0.785 |
| Total bilirubin (μmol/L) | 254.1 (193.2-304.1) | 190.4 (152.2-308.1) | 0.100 |
| Creatinine (μmol/L) | 58 (52-69) | 74 (60-87) | 0.001 |
| Sodium (mmol/L) | 138 (133-140) | 137 (132-140) | 0.741 |
| White blood cell count (10^9^/L) | 6.2 (4.4-8.22) | 5.98 (4.66-12.01) | 0.235 |
| Neutrophil count (10^9^/L) | 4.4 (3.0-6.5) | 4.5 (2.8-9.4) | 0.356 |
| Hemoglobin (g/L) | 138 (125-148) | 127 (113-138) | 0.070 |
| Platelet count (10^9^/L) | 108 (85-127) | 92 (79-113) | 0.346 |
| INR | 2.10 (1.90-2.40) | 1.85 (1.70-2.34) | 0.400 |
| LMR | 1.78 (1.32-2.74) | 1.23 (0.98-1.78) | 0.013 |
| NLR | 3.60 (2.35-8.20) | 5.36 (3.83-7.54) | 0.181 |
| MELD | 25 (24-26) | 25 (23-29) | 0.698 |

Abbreviations: MDR, multidrug-resistant; INR, international normalized ratio; LMR, lymphocyte-to-monocyte ratio; NLR, neutrophil-to-lymphocyte ratio; Data are expressed as the median (interquartile range) or percent (number).

Table 3. Characteristics of isolated bacteria in HBV-ACLF patients with bacterial infection

|  | **gram-negative bacteria**  **(*n* = 13)** | **gram-positive bacteria**  **(*n* = 6)** |
| --- | --- | --- |
| Multidrug resistant strains, % (*n*) | 5.3 (1) | 0 (0) |
| Ascitic fluid, % (*n*) | 26.3 (5) | 10.5 (2) |
| Blood, % (*n*) | 26.3 (5) | 5.3 (1) |
| Urine, % (*n*) | 10.5 (2) | 5.3 (1) |
| Sputum, % (*n*) | 10.5 (2) | 5.3(1) |

Abbreviations: HBV-ACLF, hepatitis B virus-related acute-on-chronic liver failure; Data are expressed as percent (number).
